# Theranostic Radiopharmaceuticals of Somatostatin Receptors for Patients with Neuroendocrine Tumors: Agonists Versus Antagonists—A Systematic Review and Meta-Analysis

**DOI:** 10.3390/ijms26178539

**Published:** 2025-09-02

**Authors:** Qi Wang, Damiano Librizzi, Shamim Bagheri, Ali Ebrahimifard, Azimeh Hojjat Shamami, Anja Rinke, Friederike Eilsberger, Markus Luster, Behrooz Hooshyar Yousefi

**Affiliations:** 1 Department of Nuclear Medicine, School of Medicine, Philipps Universität Marburg, Baldingerstrasse, 35043 Marburg, Germany; 2 Department of Nuclear Medicine, University Hospital OWL, Evangelisches Klinikum Bethel, 33617 Bielefeld, Germany; 3 Division of Gastroenterology and Endocrinology, Department of Internal Medicine, School of Medicine, Philipps Universität Marburg, Baldingerstrasse, 35043 Marburg, Germany

**Keywords:** neuroendocrine tumors, somatostatin receptor, agonists, antagonists

## Abstract

Neuroendocrine tumors (NETs) are a rare and heterogeneous class of neoplastic lesions, but their prevalence has increased significantly over the past three decades. These tumors are aggressive and difficult to treat. Improving diagnostic efficiency and treatment effectiveness is important for patients with neuroendocrine tumors. Radiopharmaceutical therapeutic diagnostics combines diagnosis and treatment technology and has broad prospects in precision medicine, especially for the early diagnosis and treatment of tumors. To compare the diagnostic advantages of radiolabeled somatostatin receptor agonists and antagonists for liver metastases from NETs and the disease control rate in NET patients. Systematic search of PubMed, Embase, Cochrane, Ovid, Scopus, and Web of Science databases up to 29 October 2024. Clinical trials of somatostatin receptor agonists and antagonists for NET diagnosis or treatment. Following PRISMA guidelines, data were independently extracted by two researchers. Pooled diagnostic or treatment effects and 95% CIs were reported using a random-effects meta-analysis model. Effect of somatostatin receptor agonists and antagonists in detecting liver metastases and disease control rate. Risk Ratio (RR) for liver metastasis detection and Effect Size (ES) for disease control rate were calculated. From 5291 articles, 52 were included in the meta-analysis. Radiolabeled somatostatin receptor antagonists were significantly more effective than agonists in detecting liver lesions (RR = 11.57, 95% CI: 4.10, 32.67). Disease control rates were higher with antagonists (ES = 0.90, 95% CI: 0.83, 0.96) compared to agonists (ES = 0.82, 95% CI: 0.78, 0.85, z = 2.12, *p* = 0.03). Radiolabeled somatostatin receptor antagonists outperform agonists in diagnosing hepatic lesions and controlling disease in NETs, highlighting their clinical superiority. This meta-analysis provides critical insights into the diagnostic and therapeutic efficacy of somatostatin receptor antagonists, and may offer a potential paradigm shift in the management of neuroendocrine tumors. Nevertheless, the smaller number of studies on antagonists may limit the generalizability of the findings and underscore the need for further clinical trials to validate these results.

## 1. Introduction

Neuroendocrine neoplasms (NENs) are a rare and diverse group of neoplasms originating from neuroendocrine cells throughout the body. The gastroenteropancreatic region is the most common site of origin for these tumors. The hormonal secretions of these neoplasms result in a wide spectrum of symptoms, contingent upon the tumor’s anatomic location and the specific hormone it produces [1,2,3]. NENs are classified as neuroendocrine tumors (NETs) or neuroendocrine carcinomas (NECs) depending on tumor grade, which is based on the proliferation rate, and the degree of differentiation, which is how similar the tumor is to normal cells. NETs range from inert to moderately aggressive, whereas NECs are highly aggressive. Highly differentiated NETs are classified into three categories based on mitotic rate and Ki-67 index: low-grade (G1), intermediate-grade (G2), and high-grade (G3). High-grade (G3) NETs are more aggressive than low-grade (G1) NETs. Some tumors have different levels of differentiation, called mixed neuroendocrine-nonneuroendocrine tumors [4,5]. In addition to their histological traits and anatomic sites, neuroendocrine tumors (NETs) can also be classified as functional, with these neoplasms eliciting corresponding clinical manifestations through the secretion of various biologically active compounds. Despite their relative rarity, NETs have demonstrated a notable increase in incidence over recent decades. This increase can be partially attributed to enhanced awareness and diagnostic techniques. In contrast to other types of epithelial malignancies, neuroendocrine tumors (NETs) generally appear inert; however, they may also demonstrate malignant potential, particularly when they arise in the lungs, digestive tract, and pancreas [6,7,8,9].

NETs are diagnosed and monitored through a multifaceted and comprehensive evaluation process. This process includes clinical symptoms, biochemical markers (e.g., chromogranin A, urinary 5-hydroxyindoleacetic acid, neuron-specific enolase, pancreatic polypeptides, etc.) [10], histopathology, and imaging (e.g., anatomical imaging (CT, MRI, etc.).); functional imaging ([^68^Ga]Ga-DOTA-SSA PET/CT/MRI and [^18^F]F-FDG PET/CT); and molecular genetic testing (testing for mutations such as MEN1 and TP53). These tools, along with emerging technologies like NETest and liquid biopsies (testing for circulating tumor DNA and microRNA), improve diagnostic accuracy and disease monitoring efficiency as well as precision medicine in the treatment of NENs [11,12,13].

The treatment of NETs is individualized according to the biology of the tumor and is flexible depending on the biology of the tumor and the extent of the lesion. However, for resectable disease, the first recommended procedure is resection with regional lymph node dissection. For biologically abnormal disease, neoadjuvant chemotherapy may be performed on a case-by-case basis. Somatostatin Analogues are recommended for patients who are SSR-positive or have hormonal symptoms. Other recommended treatments include peptide receptor radionuclide therapy (PRRT), targeted therapy, immunotherapy, chemotherapy, and liver-directed therapy. Palliative radiotherapy is recommended for patients with symptomatic bone metastases [4]. In recent years, PRRT has rapidly evolved into a more precise and highly targeted treatment modality. Remarkable potential has been shown by targeted radiation therapy, both in terms of efficacy and safety [14].

**Figure 1:**
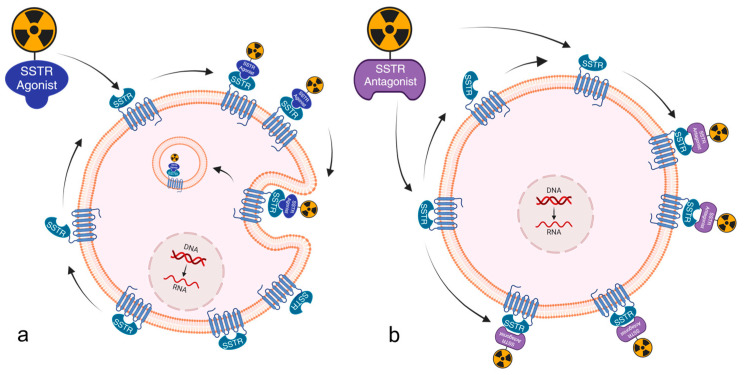
The utilization of radiolabeled Somatostatin Receptor (SSTR) agonists and antagonists has been demonstrated to facilitate imaging and therapeutic applications by binding to SSTR, a G-protein-coupled receptor that is overexpressed on neuroendocrine tumor cells. (**a**) The interaction of radiolabeled SSTR agonists with activated SSTR structures results in receptor internalization. (**b**) Radiolabeled SSTR antagonists interact with both activated and inactivated SSTR structures; however, they do not induce receptor internalization. The accumulation of radioactivity within tumor cells can be leveraged to generate imaging signals and enhance the efficacy of radiation therapy. Antagonists cause more radionuclides to accumulate on the surface of tumor cells, producing a stronger imaging signal and enhancing the effects of radiation therapy.

**Figure 2:**
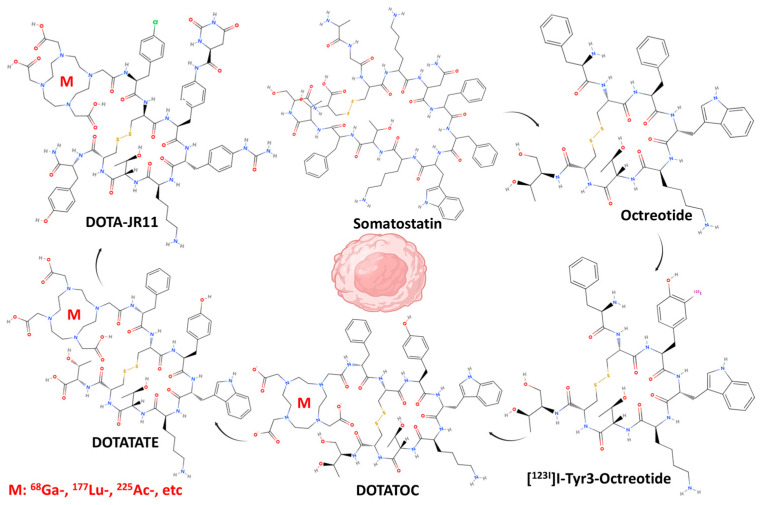
Examples are provided for the development of agonist and antagonist radiopharmaceuticals for diagnostic and therapeutic use, based on somatostatin receptors, and their evolution. Also provided are the chemical structures of the compounds.

Somatostatins represent a class of peptide hormones that exist in two natural forms: somatostatin-14 (comprising 14 amino acids) and somatostatin-28 (comprising 28 amino acids). These hormones exhibit a high affinity for five specific subtypes of the somatostatin receptor (SSTR1, SSTR2, SSTR3, SSTR4, and SSTR5) [15]. The expression levels of these receptors vary significantly between normal physiological tissues and tumor samples, with higher levels of expression observed in tumors. The high expression levels of somatostatin receptors in tumors have led to the development of radiolabeled somatostatin receptor-based treatments and diagnostic methods for the management and evaluation of tumors [16,17]. The utilization of diagnostic and therapeutic radiopharmaceuticals (a pharmaceutical containing a radioactive isotope (radionuclide)), which are based on somatostatin receptor agonists, has been extensively promoted in clinical practice for the treatment of neuroendocrine tumors. These radiopharmaceuticals have been shown to exert a pivotal role in enhancing patient prognosis and quality of life [18,19,20,21,22,23]. A critical consideration in the application of agonists (compounds that bind to a receptor and activate it) for the management of neuroendocrine tumors pertains to their capacity for effective internalization within the tumor cells. Upon high-affinity binding to the receptor, agonists typically induce internalization of the ligand-receptor complex and promote accumulation of radiolabel. This process contributes to an efficient radiotherapeutic effect inside tumor cells while enhancing the intensity of the imaging signal, thus improving diagnostic and therapeutic accuracy [16,24,25]. Recently introduced SSTR antagonists, such as [^68^Ga]Ga-DOTA-JR11 [26], have made significant advances in the field of SSTR targeting. Preclinical and clinical studies have demonstrated that radiolabeled SSTR antagonists exhibit superior pharmacokinetics and tumor-to-background ratios (TBR) compared to somatostatin analogs (SSA) [27]. This enhanced performance may be attributed to their higher affinity for SSTR, despite the absence of internalization induction capacity in antagonists. Contrary to agonists, antagonists (compounds that bind to receptor but block or dampen the biological response that would normally be triggered by an agonist) do not promote internalization, and they bind to both the activated and inactivated conformations of SSTR, resulting in a slower dissociation rate compared to agonists. Consequently, the accumulation of radiolabel on the tumor cell surface is more intense compared to agonists. Furthermore, SSTR antagonists have a prolonged duration of action and enhanced stability in hydrophobic environments due to their enhanced chemical stability and hydrophobicity. In conclusion, SSTR antagonists exhibit excellent pharmacokinetic properties and better tumor visualization (Figure 1 and Figure 2) [28,29,30].

In this meta-analysis, we aimed to compare the diagnostic advantages of radiolabeled somatostatin receptor agonists and antagonists for liver metastases from neuroendocrine tumors and the disease control rate in patients with neuroendocrine tumors.

## 2. Materials and Methods

Systematic reviews and meta-analyses were conducted in accordance with the guidelines set forth by the Cochrane Collaboration, and the results were reported in adherence to the PRISMA (Preferred Reporting Items for Systematic Evaluations and Meta-Analyses) reporting guidelines. The meta-analysis was registered in the International Register of Protocols for Systematic Evaluation and Meta-Analysis (INPLASY2024120022). As this study did not involve human participants, no institutional review board approval or informed consent was required.

A systematic search of the PubMed, Embase, Cochrane, Ovid, Scopus, and Web of Science databases was conducted from inception to 29 October 2024. The search terms are detailed in Table A1. Following the removal of duplicates, two reviewers undertook a screening of the titles and abstracts. Additionally, the reference lists of the included studies and other published meta-analyses were reviewed. The full texts of the articles were evaluated independently by two reviewers, and the literature was screened in accordance with predefined criteria. Any discrepancies were resolved through negotiation. In the event that consensus could not be reached through discussion, a third reviewer was consulted to determine a resolution. Studies were considered eligible if they met the following criteria: (1) patients 18 years of age or older with neuroendocrine tumors; (2) radiolabeled somatostatin receptor agonists or antagonists; (3) diagnostic or therapeutic studies; (4) assessment of the disease control rate according to the RECIST1.1 criteria; (5) assessment of diagnostic efficacy for liver metastases; (6) patient-based studies; (7) non-combination therapy. Two authors independently extracted data using a standardized, predefined data collection form. Inconsistencies in the data were compared and combined into a final dataset that was independently checked by two additional reviewers. Articles were excluded if the patients in the study had tumors other than neuroendocrine tumors or if the extracted data were not suitable for meta-analysis. Non-English studies were also excluded. The outcome metrics were to compare the detection advantage of liver metastases and disease control rate in neuroendocrine tumors by radiotracer somatostatin receptor agonists versus antagonists. Weighted combined treatment effects were calculated using a random effects model. The variability between studies due to heterogeneity was estimated using the *I*^2^ statistic, with values greater than 50% indicating significant heterogeneity. Publication bias was comprehensively assessed using funnel plots, the Begg’s test, and the Egger’s test. Data analysis was performed with STATA 18 (StataCorp LLC, College Station, TX, USA).

## 3. Results

**Figure 3:**
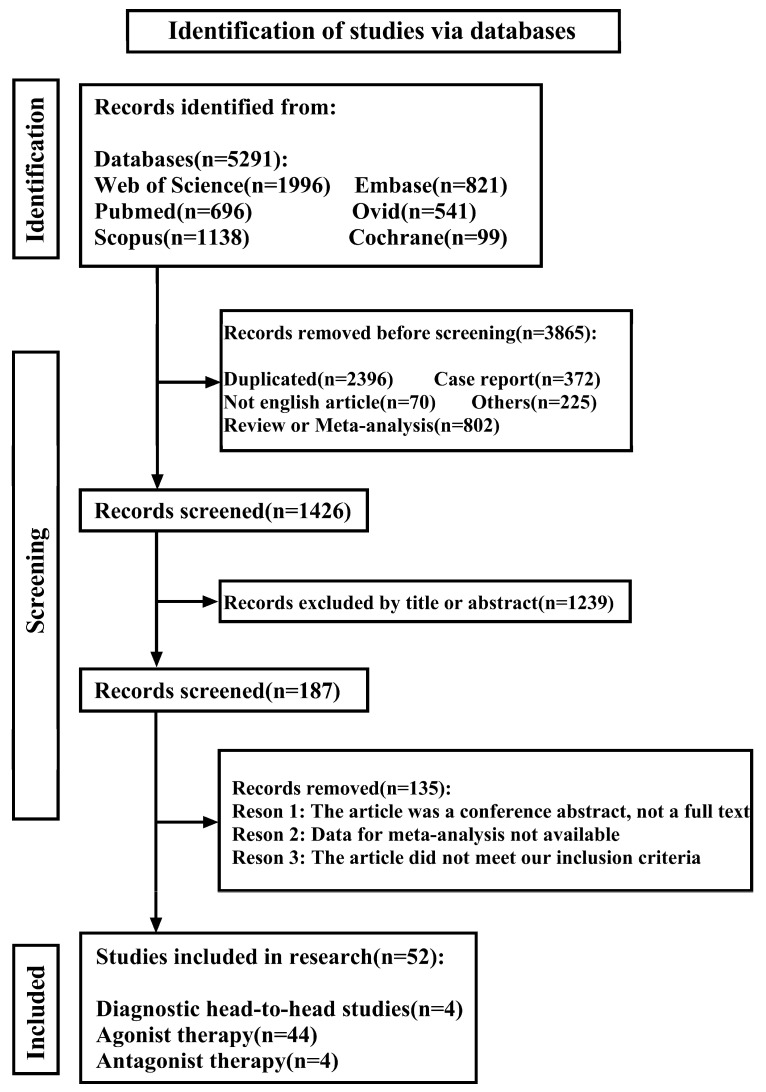
Literature screening flow chart.

**Figure A1:**
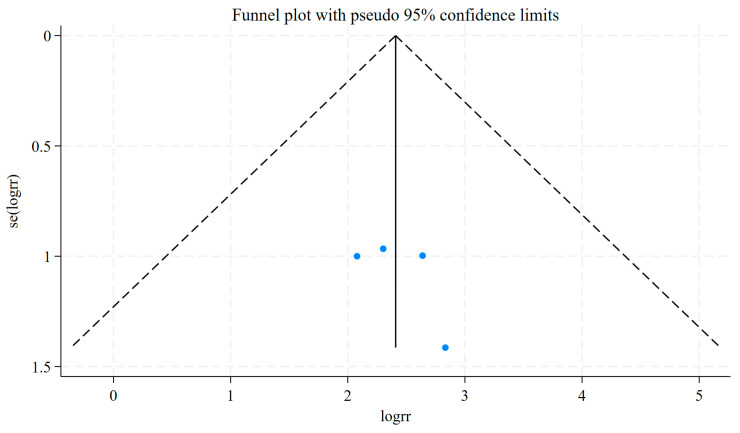
Head-to-head comparison of the detection rate of liver metastases between radiolabeled somatostatin receptor agonists and antagonists in patients with neuroendocrine tumors. The pooled results of the funnel plot. logrr: log risk ratio, se(logrr): standard error of the log risk ratio.

**Figure A2:**
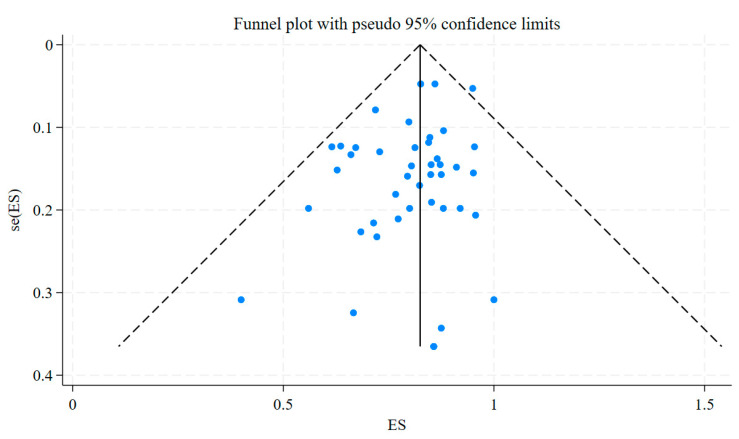
Disease control rates with radiolabeled somatostatin receptor agonists in patients with neuro-endocrine tumors. The pooled results of the funnel plot. ES: effect size, se (ES): standard error of the effect size.

**Figure A3:**
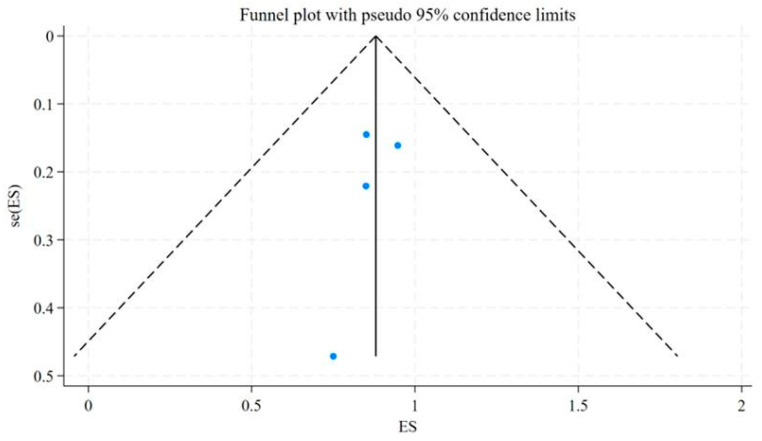
Disease control rates with radiolabeled somatostatin receptor Antagonists in patients with neuroendocrine tumors. The pooled results of the funnel plot. ES: effect size, se (ES): standard error of the effect size.

We ultimately collected 5291 papers, resulting in the final inclusion of 52 papers that could be used for meta-analysis (please see Figure 3 for the literature screening process). Among these, 4 articles were head-to-head comparisons of antagonists versus agonists effectiveness in detecting liver metastases in neuroendocrine tumors [31,32,33,34], 44 were used to pool agonist-treated neuroendocrine tumor disease control rates [35,36,37,38,39,40,41,42,43,44,45,46,47,48,49,50,51,52,53,54,55,56,57,58,59,60,61,62,63,64,65,66,67,68,69,70,71,72,73,74,75,76,77,78], and a further 4 articles were used to pool antagonist-treated neuroendocrine tumor disease control rates [30,79,80,81]. Four articles on diagnosis were tested for publication bias, and the results suggested that the funnel plot was asymmetric by Begg’s Test *p* = 0.462; meanwhile, Egger’s test *p* = 0.016, so we excluded the literature of Lin, Z. et al. [33] due to its unavailability. Finally, three articles were pooled and analyzed, and the pooled results of the funnel plot were symmetric (Figure A1), while Begg’s Test *p* = 0.734 and Egger’s Test *p* = 0.370. Regarding treatment, we did not find publication bias in the pooling of 44 studies of agonists for neuroendocrine tumors, with Begg’s Test *p* = 0.491 and Egger’s test *p* = 0.061, and the funnel plot was symmetrical (Figure A2). No publication bias was found after pooling the four studies of antagonists for neuroendocrine tumors, Begg’s Test *p* = 0.734, Egger’s test *p* = 0.494, and the funnel plot was symmetrical (Figure A3).

### 3.1. Diagnosis

**Figure 4:**
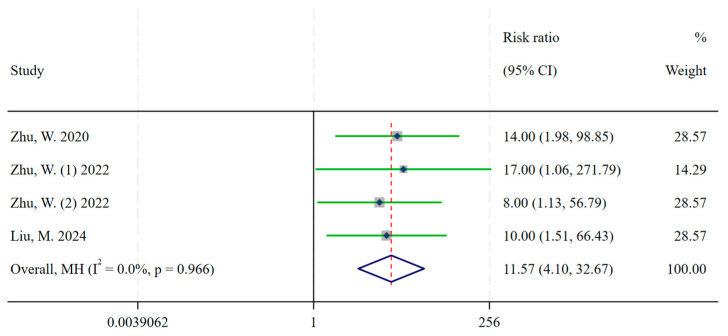
Head-to-head comparison of the detection rate of liver metastases between radiolabeled somatostatin receptor agonists and antagonists in patients with neuroendocrine tumors. The pooled results were RR = 11.57, (95% CI: 4.10, 32.67) [31,32,34]. Note: RR is risk ratio, weights are from Mantel-Haenszel model (MH); continuity correction applied to studies with zero cells.

In three articles included 4 head-to-head clinical trials (140 participants), with 4 antagonists and 1 agonist SST radiopharmaceuticals. The antagonists were significantly superior to agonists in the detection of liver metastases (RR = 11.57, 95% CI: 4.10–32.67) (Figure 4 and Table A2). There was no significant heterogeneity between studies (*I*^2^ = 0.0%, *p* = 0.966).

### 3.2. Therapy

**Figure 5:**
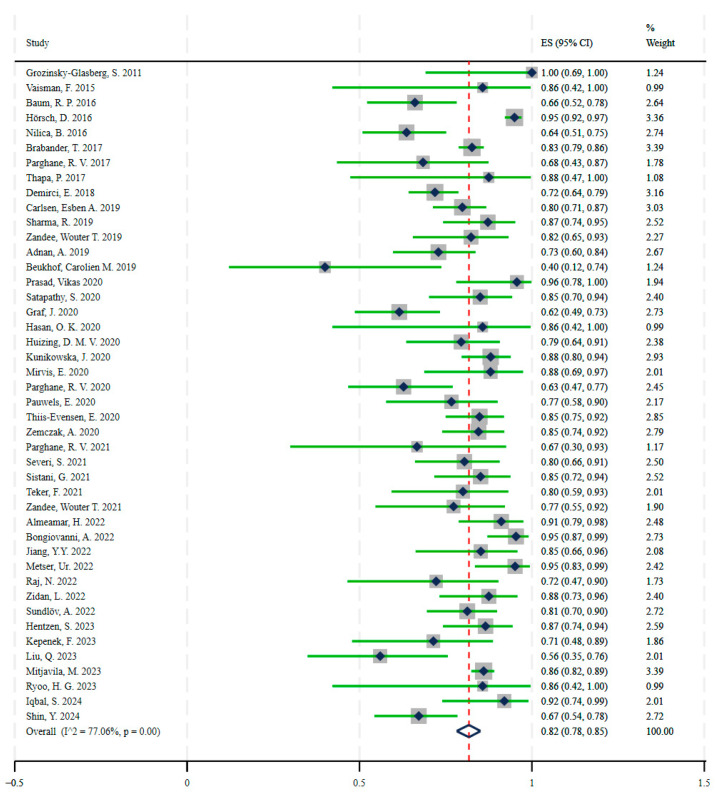
Disease control rates with radiolabeled somatostatin receptor agonists in patients with neuroendocrine tumors. The pooled result was ES = 0.82, (95% CI: 0.78, 0.85) [35,36,37,38,39,40,41,42,43,44,45,46,47,48,49,50,51,52,53,54,55,56,57,58,59,60,61,62,63,64,65,66,67,68,69,70,71,72,73,74,75,76,77,78].

**Figure 6:**
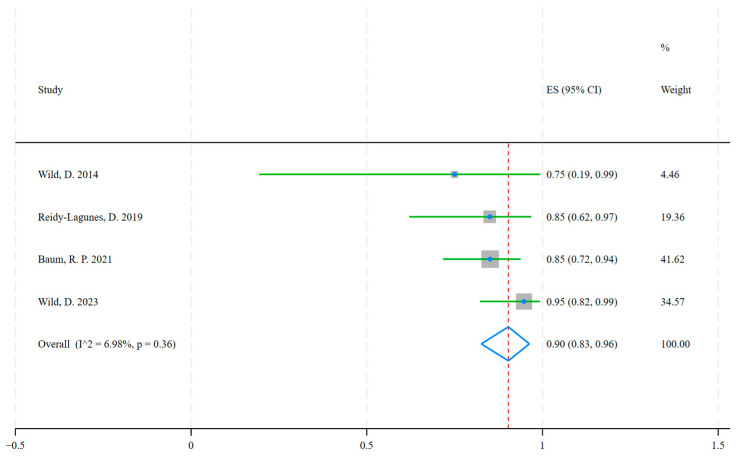
Disease control rates with radiolabeled somatostatin receptor Antagonists in patients with neuroendocrine tumors. The pooled result was ES = 0.90, (95% CI: 0.83, 0.96) [30,79,80,81].

A total of 44 agonist therapy trials with 2990 patients and 4 antagonist treatment trials with 109 patients were included. In the patient-based studies, the disease control rate was ES = 0.82 (95% CI: 0.78, 0.85), test of ES = 0: z = 47.49, *p* = 0.00, for agonist-treated NETs (Figure 5) (Table A3) and ES = 0.90 (95% CI: 0.83, 0.96), test of ES = 0: z = 21.23, *p* = 0.00, for antagonist-treated NETs (Figure 6) (Table A4). The Z-test showed that the antagonist outperformed the agonist in terms of outcome, with a Z-value = 2.12 and *p* = 0.03, indicating that the disease control rate of the antagonist (ES = 0.90) was significantly higher than that of the agonist (ES = 0.82). There was no significant heterogeneity between antagonist treatment trials (*I*^2^ = 6.98%, *p* = 0.36), whereas we demonstrated heterogeneity between agonist treatment trials (*I*^2^ = 77.06%, *p* = 0.00).

## 4. Discussion

To our knowledge, this is the first meta-analysis of somatostatin receptor agonists and antagonists for diagnosing and treating neuroendocrine tumors. Theranostics (also spelled theragnostics), which is a blend of therapy and diagnostics, is an important component of modern nuclear medicine. It enables precise diagnosis and treatment of tumors by identifying disease-specific characteristics, and integrates diagnosis and treatment using the same molecular targets, embodying the concept of ‘diagnostic and therapeutic integration’ [82].

The diagnostic and therapeutic management of neuroendocrine tumors should include monitoring clinical symptoms, assessing biochemical parameters, and performing routine and SSR imaging examinations [83]. Radiopharmaceuticals of SSTRs can achieve ‘integrated diagnosis and treatment’ and have broad application prospects for patients with advanced NETs [84]. [^68^Ga]Ga-DOTA-SSA has been shown to offer advantages over other diagnostic methods, particularly in patients with SSR-positive disease [85]. Somatostatin receptor radioligands have received sustained attention from a wide range of scientific researchers, greatly contributing to the development of peptide radiopharmaceuticals [86,87]. [^68^Ga]-based tracers, such as [^68^Ga]Ga-DOTA-TATE, are well-established and reliable. Additionally, [^18^F]-labeled SSR tracers, including [^18^F]F-SiFAlin-TATE, demonstrate significant potential. When PET/CT is unavailable, planar SSR scintigraphy ([^111^In]In-DTPA-octreotide scintigraphy), SPECT/CT ([^99m^Tc]Tc-Tektrotyd SPECT/CT) or [^123^I-]I-MIBG scintigraphy as alternative methods, due to their lower spatial resolution and diagnostic accuracy, higher radiation dose, and longer procedure duration. SSR imaging is also important for pre-treatment assessment in patients with multiple metastatic tumors undergoing peptide receptor radionuclide therapy (PRRT) [83,88]. The NETTER-1 study demonstrated that PRRT is well-tolerated and effective for patients with unresectable or metastatic, well-differentiated NETs who have experienced tumor progression under SSA biological therapy [89]. Additionally, [^18^F]F-FDG-PET/CT may be beneficial for patients with NET G2 or G3. The role of CT and MRI scans should not be overlooked, as they are widely used and provide consistent results [83]. The application of somatostatin receptor radiopharmaceuticals in clinical practice has undoubtedly improved the diagnostic and therapeutic efficacy of patients with neuroendocrine tumors, as well as the quality of life and survival rate of patients [90,91]. Since antagonists can bind to more tumor targets than agonists, a paradigm shift in binding from internalizing SSTR2 agonists to antagonists is feasible [29,92,93]. Therefore, antagonists have a superior clinical indication.

Our study with three articles including 4 head-to-head clinical trials of agonists versus antagonists found that antagonists showed superior detection of hepatic metastatic compared with agonists in patient-based comparisons (RR = 11.57, 95% CI: 4.35, 32.67) and that this advantage may be due to antagonists having more binding sites at the SSTR receptor, as well as lower hepatic background uptake of antagonists compared to agonists in patients with neuroendocrine tumors. The majority of current antagonists have good clinical performance, especially in normal tissues where uptake is significantly lower than that of agonists. Krebs, S et al. [94] in a biodistribution and metrological analysis study of the antagonist [^68^Ga]Ga-DOTA-JR11 in patients with metastatic neuroendocrine tumors confirmed that the image contrast of liver lesions was significantly higher with [^68^Ga]Ga-DOTA-JR11 PET/CT compared to [^68^Ga]Ga-DOTA-TATE. They attributed this finding primarily to the fact that normal liver parenchyma has a much lower uptake on [^68^Ga]Ga-DOTA-JR11 PET/CT, making it easier to detect liver metastases. The liver represents a principal site of metastasis in patients with NETs [95], and the low background activity of the antagonist in the liver provides optimal image contrast. This capacity is of paramount importance not only for the diagnosis of NETs but also for the formulation of appropriate therapeutic strategies. The capacity to discern supplementary liver lesions through the utilization of antagonists may also result in a modification of the treatment plan when the prospect of localized treatment of liver metastases is contemplated. The performance of partial hepatectomy is questioned or even in instances where supplementary or bilobar liver lesions are identified [32,96].

In this analysis, 44 studies of radiolabeled somatostatin receptor agonists reported disease control rates for agonists in neuroendocrine tumors of ES = 0.82 (95% CI: 0.78, 0.85), z = 47.49, *p* = 0.00, and four studies of radiolabeled somatostatin receptor antagonists reported disease control rates for antagonists in neuroendocrine tumors of ES = 0.90 (95% CI: 0.83, 0.96), z = 21.23, *p* = 0.00. Comparison of the disease control rate of agonists and antagonists in neuroendocrine tumors by Z-test (z = 2.12, *p* = 0.03) showed that antagonists may be superior to agonists in the treatment of neuroendocrine tumors. SST2 receptor antagonists have favorable pharmacokinetic and biodistribution profiles, such as longer intratumoral residence time and higher tumor uptake, compared to agonists, and at the same time, antagonists have a higher tumor dose than agonists. In a preliminary clinical study of four patients with progressive NETs by Wild et al. [30], the tumor dose of the SSTR antagonist [^177^Lu]Lu-DOTA-JR11 was 1.7 to 10.6 times higher than that of the agonist [^177^Lu]Lu-DOTATATE. Despite the favorable results of clinical trials with antagonists, we cannot ignore the potentially toxic side effects, and in a study by Wild, D. et al. [81] on [^177^Lu]Lu-satoreotide tetraxetan, the radioactivity of radiopharmaceutical administered had to be reduced due to hematological toxicity.

A limitation of this study is the inclusion of a smaller number of scientific publications about antagonists, due to the fact that there are fewer clinical studies on the diagnosis and treatment using radiolabeled somatostatin receptor antagonists, which may have an impact on the generalizability of the conclusions. Furthermore, we included as many articles as possible on agonist treatment of neuroendocrine tumors in order to perform a comprehensive analysis. This resulted in considerable heterogeneity among the included studies, which may be attributed to the fact that the literature was included without specific limitations in terms of study design. In addition, the number of patients included in the respective studies and the duration of follow-up varied widely, as did the types of neuroendocrine tumors and the types of radiopharmaceuticals involved. However, despite the heterogeneity, the combined effect size was significantly different from zero (ES = 0: z = 47.49, *p* = 0.00), indicating a strong overall effect. The study by Wild D. et al. [30] in our analysis had only 4 patients. Although this study included only 4 patients, it provides the first clinical evidence that radiolabeled SST antagonists were superior to SST agonists in treating neuroendocrine tumors. Also, despite the small sample size, the results are in good accordance with other studies, suggesting that the conclusions drawn from this study are valid. It should be noted that this review did not include studies examining the use of PRRT in combination with chemotherapeutic agents for the treatment of neuroendocrine tumors. The articles by Huizing, D. M. V. et al. [53] and Kunikowska, J. et al. [54] both evaluated DCR at two time points. To avoid data duplication, we only included the DCR results with a longer follow-up time in our study. Additionally, studies of retreatment with PRRT (R-PRRT) after PRRT or re-retreatment with PRRT (RR-PRRT) after R-PRRT were not included. Several articles utilizing actinium-225 were also excluded on the grounds that alpha-particle rays are considered to be more distinct from beta particles.

## 5. Conclusions

The present study found that radiolabeled somatostatin receptor antagonists show significant advantages over agonists in detecting liver metastases and controlling disease in neuroendocrine tumor patients. The meta-analysis found that antagonists were significantly more effective in detecting liver lesions (RR = 11.57, 95% CI: 4.10, 32.67). Moreover, it had higher disease control rates (antagonist ES = 0.90, 95% CI: 0.83, 0.96) compared to agonists (agonist ES = 0.82, 95% CI: 0.78, 0.85), the z-value was 2.12, and the *p*-value was 0.03. This meta-analysis provides critical insights into the diagnostic and therapeutic efficacy of somatostatin receptor antagonists, and may offer a potential paradigm shift in the management of neuroendocrine tumors.

These findings highlight the potential of antagonists to improve diagnostic efficiency and treatment outcomes, emphasizing the importance of further research in this area to enhance patient care in NETs. Nevertheless, the smaller number of studies on antagonists may limit the generalizability of the findings and underscore the need for further clinical trials to validate these results.

## Data Availability

All data are included in the article. If further information is required, please contact the corresponding author.

## Abbreviations

NENs	Neuroendocrine neoplasms
NETs	Neuroendocrine tumors
NECs	Neuroendocrine carcinomas
RR	Risk Ratio
ES	Effect Size
SSTR	Satostatin Receptor
PRRT	Peptide receptor radionuclide therapy
PRISMA	Preferred Reporting Items for Systematic Evaluations and Meta-Analyses
TBR	Tumor-to-Background Ratios
SSA	Somatostatin Analogs
MR	Minor Response
CR	Complete Response
PR	Partial Response
SD	Stable Disease
DCR	Disease Control Rate
MH	Mantel-Haenszel model

## Appendix A

**Table A1:** Literature search strategy (taking PubMed as an example).

((((“Neuroendocrine Tumors”[Mesh]) OR (((((Neuroendocrine Tumor[Title/Abstract]) OR (Tumor, Neuroendocrine[Title/Abstract])) OR (Tumors, Neuroendocrine[Title/Abstract])) OR (NETs[Title/Abstract])) OR (NET[Title/Abstract]))) AND ((“Receptors, Somatostatin”[Mesh]) OR ((((((Somatostatin Receptor[Title/Abstract]) OR (Receptor, Somatostatin[Title/Abstract])) OR (Somatostatin Receptors[Title/Abstract])) OR (Receptors, Somatotropin Release Inhibiting Hormone[Title/Abstract])) OR (Receptors, SRIH[Title/Abstract])) OR (SRIH Receptors[Title/Abstract])))) AND ((((((((((“agonists” [Subheading]) OR (“antagonists and inhibitors” [Subheading])) OR (((antagonists[Title/Abstract]AND inhibitors[Title/Abstract]) OR (antagonists[Title/Abstract])) OR (inhibitors[Title/Abstract]))) OR ((“gallium Ga 68 DOTATATE” [Supplementary Concept]) OR ((((((((((((((DOTATATE gallium ga-68[Title/Abstract]) OR (gallium 68 DOTA-octreotide[Title/Abstract])) OR (gallium (68ga) dota-tate[Title/Abstract])) OR (gallium-dota-octreotate, ga-68[Title/Abstract])) OR (gallium DOTATATE, ga-68[Title/Abstract])) OR (gallium 68 DOTATATE[Title/Abstract])) OR (68Ga-DOTATATE[Title/Abstract])) OR (68gallium-DOTA-Tyr(3)-Thr(8)-octreotate[Title/Abstract])) OR (edotreotide gallium ga-68[Title/Abstract])) OR (gallium ga-68 edotreotide[Title/Abstract])) OR (gallium edotreotide ga-68[Title/Abstract])) OR (gallium ga 68-dotatoc[Title/Abstract])) OR (Ga-68 dota0-tyr3-octreotide[Title/Abstract])) OR (gallium Ga 68-edotreotide[Title/Abstract])))) OR ((“68Ga-DOTANOC” [Supplementary Concept]) OR (68Ga-DOTA-NOC[Title/Abstract]))) OR ((“Ga(III)-DOTATOC” [Supplementary Concept]) OR (((67Ga-DOTATOC[Title/Abstract]) OR (gallium-68 DOTATOC[Title/Abstract])) OR (68Ga-DOTATOC[Title/Abstract])))) OR ((“lutetium Lu 177 DOTATATE” [Supplementary Concept]) OR ((((((((177lutetium-DOTA-O-Tyr3-octreotate[Title/Abstract]) OR (lutetium 177Lu oxodotreotide[Title/Abstract])) OR (Lu-177 DOTATE[Title/Abstract])) OR (177Lu-DOTAOTyr3-octreotate[Title/Abstract])) OR (DOTATATE-177Lu[Title/Abstract])) OR (177Lu-DOTATATE[Title/Abstract])) OR (lutetium oxodotreotide Lu-177[Title/Abstract])) OR (Lutathera[Title/Abstract])))) OR ((“177Lu-octreotide, DOTA(0)-Tyr(3)-” [Supplementary Concept]) OR ((177Lu-octreotide, DOTA0, tyrosyl3-[Title/Abstract]) OR (177Lu-DOTATOC[Title/Abstract])))) OR ((((((68Ga-DOTA-JR11[Title/Abstract]) OR (18F-AlF-NOTA-LM3[Title/Abstract])) OR (68Ga-OPS202[Title/Abstract])) OR (68Ga-NODAGA-JR11[Title/Abstract])) OR (111In-DTPA-octreotide[Title/Abstract])) OR (68Ga-DATA(5m)-LM4[Title/Abstract]))) OR (((((177Lu-DOTA-JR11[Title/Abstract]) OR (177Lu-DOTA-LM3[Title/Abstract])) OR (tetulomab tetraxetan lu-177[Title/Abstract])) OR (177LU-DOTA-HH1[Title/Abstract])) OR (177Lu-Satoreotide Tetraxetan[Title/Abstract])))) AND ((((Theranostics[Title/Abstract]) OR (Theranostic[Title/Abstract])) OR ((“Therapeutics”[Mesh]) OR (((((Therapeutic[Title/Abstract]) OR (Therapy[Title/Abstract])) OR (Therapies[Title/Abstract])) OR (Treatment[Title/Abstract])) OR (Treatments[Title/Abstract])))) OR ((“Diagnosis”[Mesh]) OR ((((((((((((((Diagnoses[Title/Abstract]) OR (Diagnose[Title/Abstract])) OR (Diagnoses[Title/Abstract]AND Examinations[Title/Abstract])) OR (Diagnoses[Title/Abstract]AND Examination[Title/Abstract])) OR (Examination[Title/Abstract]AND Diagnoses[Title/Abstract])) OR (Examinations[Title/Abstract]AND Diagnoses[Title/Abstract])) OR (Antemortem Diagnosis[Title/Abstract])) OR (Antemortem Diagnoses[Title/Abstract])) OR (Diagnoses, Antemortem[Title/Abstract])) OR (Diagnosis, Antemortem[Title/Abstract])) OR (Postmortem Diagnosis[Title/Abstract])) OR (Diagnoses, Postmortem[Title/Abstract])) OR (Diagnosis, Postmortem[Title/Abstract])) OR (Postmortem Diagnoses[Title/Abstract]))))

**Table A2:** Head-to-head comparison of the detection rate of liver metastases between radiolabeled somatostatin receptor agonists and antagonists in patients with neuroendocrine tumors.

Study	Year	Country	Neuroendocrine Tumor Types	Event_Exp ^1^	Total_Exp ^2^	Radiopharmaceuticals_Exp	Event_Ctrl ^3^	Total_Ctrl ^4^	Radiopharmaceuticals_Ctrl
Zhu, W.	2020	China	Metastatic, Well-Differentiated NETs	14	26	[^68^Ga]Ga-DOTA-JR11	1	26	[^68^Ga]Ga-DOTATATE
Zhu, W. (1)	2022	China	Well-differentiated NETs	8	16	[^68^Ga]Ga-NODAGA-LM3	0	16	[^68^Ga]Ga-DOTATATE
Zhu, W. (2)	2022	China	Well-differentiated NETs	8	16	[^68^Ga]Ga-DOTA-LM3	1	16	[^68^Ga]Ga-DOTATATE
Liu, M.	2024	China	Well-differentiated NETs	10	12	[^18^F]AlF-NOTA-LM3	1	12	[^68^Ga]Ga-DOTATATE

^1^ Event_Exp: The number of patients with more liver metastatic lesions detected with antagonists than with agonists. ^2^ Total_Exp: Number of all patients tested for antagonists. ^3^ Event_Ctrl: The number of patients with more liver metastatic lesions detected with agonists than with antagonists. ^4^ Total_Ctrl: Number of all patients tested for agonists.

**Table A3:** Disease control rates with radiolabeled somatostatin receptor agonists in patients with neuroendocrine tumors.

Study	Year	Country	Event (DCR = CR + PR + SD) ****	Total	Neuroendocrine Tumor Types	Radiopharmaceuticals
Grozinsky-Glasberg, S.	2011	Israel	10	10	Malignant Gastrinomas	[^90^Y]Y/[^177^Lu]Lu-DOTATOC
Vaisman, F.	2015	Brazil	6	7	Medullary Thyroid Cancer	[^177^Lu]Lu-DOTATATE
Baum, R. P.	2016	Germany	37	56	Gastroenteropancreatic and other NETs	[^177^Lu]Lu-DOTATOC
Hörsch, D.	2016	Germany	339	357	Gastroenteropancreatic, Bronchial, and other NETs	[^177^Lu]Lu/[^90^Y]Y-DOTATOC/DOTATATE
Nilica, B.	2016	Austria	42	66	Gastroenteropancreatic, Pulmonary, and other NETs	[^90^Y]Y-DOTATOC/[^177^Lu]Lu-DOTATATE
Brabander, T. **	2017	Netherlands	366	443	Gastroenteropancreatic and Bronchial NETs	[^177^Lu]Lu-DOTATATE
Parghane, R. V. *	2017	India	13	19	Pulmonary NETs	[^177^Lu]Lu-DOTATATE
Thapa, P.	2017	India	7	8	Thymu, Mediastinum, Ureter, Esophagus, and Sacral NETs	[^177^Lu]Lu-DOTATATE
Demirci, E.	2018	Turkey	115	160	Bronchial, Pancreatic, Nonpancreatic Gastroenteropancreatic-NETs, Pheochromocytoma–Paraganglioma, and other NETs	[^177^Lu]Lu-DOTATATE
Carlsen, Esben A.	2019	Europe	91	114	Gastroenteropancreatic and other NETs	[^177^Lu]Lu/[^90^Y]Y/[^111^In]In-DOTATOC/DOTATATE
Sharma, R.	2019	England	41	47	Gastroenteropancreatic and other NETs	[^177^Lu]Lu-DOTATATE
Zandee, Wouter T.	2019	Netherlands	28	34	Pancreatic NETs	[^177^Lu]Lu-DOTATATE
Adnan, A.	2019	India	43	59	Gastroenteropancreatic, lung, other, and unknown	[^177^Lu]Lu-DOTATATE
Beukhof, Carolien M.	2019	Netherlands	4	10	Medullary thyroid carcinoma (MTC)	[^177^Lu]Lu-octreotate
Prasad, Vikas	2020	Europe, USA	22	23	Gastroenteropancreatic and lung-NETs	[^177^Lu]Lu-DOTATATE/DOTATOC
Satapathy, S.	2020	India	34	40	Gastroenteropancreatic, lung, paraganglioma, MTC, and unknown	[^177^Lu]Lu-DOTATATE
Graf, J.	2020	Germany	40	65	Gastroenteropancreatic, pulmonary, and other NETs	[^177^Lu]Lu-DOTATOC/[^177^Lu]Lu-DOTATATE
Hasan, O. K.	2020	Canada, Australia, Israel	6	7	Esthesioneuroblastoma	[^111^In]In/[^90^Y]Y/[^177^Lu]Lu-octreotide
Huizing, D. M. V.	2020	Netherlands	31	39	Gastroenteropancreatic and Pulmonary NETs	[^177^Lu]Lu-DOTATATE
Kunikowska, J.	2020	Poland	81	92	Gastroenteropancreatic, Pulmonary, and other NETs	[^90^Y]Y/[^177^Lu]Lu-DOTATATE
Mirvis, E.	2020	England	22	25	Bronchial NETs	[^90^Y]Y/[^177^Lu]Lu-DOTATATE
Parghane, R. V.	2020	India	27	43	Medullary thyroid carcinoma	[^177^Lu]Lu-DOTATATE
Pauwels, E.	2020	Europe	23	30	Gastroenteropancreatic and other NETs	[^90^Y]Y-DOTATOC
Thiis-Evensen, E.	2020	Norway	67	79	Gastroenteropancreatic, pulmonary, and other NETs	[^177^Lu]Lu-DOTA-octreotate
Zemczak, A.	2020	Poland	60	71	Gastroenteropancreatic, Pulmonary, and other NETs	[^90^Y]Y/[^177^Lu]Lu-DOTATATE
Parghane, R. V. *	2021	India	6	9	Paraganglioma	[^177^Lu]Lu-DOTATATE
Severi, S.	2021	Italy	37	46	Pheochromocytoma and Paraganglioma	[^90^Y]Y-DOTATOC/[^177^Lu]Lu-DOTATATE
Sistani, G.	2021	Canada	40	47	Gastroenteropancreatic, Pulmonary, and other NETs	[^177^Lu]Lu-DOTATATE
Teker, F.	2021	Turkey	20	25	Gastroenteropancreatic NETs	[^177^Lu]Lu-DOTATATE
Zandee, Wouter T.	2021	Netherlands	17	22	Midgut NETs	[^177^Lu]Lu-DOTATATE
Almeamar, H.	2022	Ireland, Sweden, England	41	45	Gastroenteropancreatic, Pulmonary, and other NETs	[^177^Lu]Lu-DOTA-octreotate
Bongiovanni, A.	2022	Italy	62	65	Gastroenteropancreatic, Pulmonary, and other NETs	[^177^Lu]Lu-DOTATATE
Jiang, Y.Y.	2022	China, Singapore	23	27	Gastroenteropancreatic, Paraganglioma, and otherNETs	[^177^Lu]Lu-DOTA-EB-TATE PRRT
Metser, Ur.	2022	Canada	39	41	Gastroenteropancreatic, Pulmonary, and other NETs	[^177^Lu]Lu-DOTATATE
Raj, N.	2022	United States	13	18	Gastroenteropancreatic, Pulmonary, and other NETs	[^177^Lu]Lu-DOTATATE
Zidan, L.	2022	Australia, United States, Israel, England	35	40	Pulmonary NETs	[^177^Lu]Lu-DOTATATE
Sundlöv, A.	2022	Sweden	52	64	Gastroenteropancreatic and other NETs	[^177^Lu]Lu-DOTATATE
Hentzen, S.	2023	United States	45	52	Gastroenteropancreatic NETs	[^177^Lu]Lu-DOTATATE
Kepenek, F.	2023	Turkey	15	21	Gastroenteropancreatic NETs	[^177^Lu]Lu-DOTATATE
Liu, Q.	2023	Germany, China	14	25	Medullary thyroid carcinoma	[^90^Y]Y/[^177^Lu]Lu-DOTATATE/DOTATOC/DOTANOC
Mitjavila, M.	2023	Spain	381	443	Gastroenteropancreatic, Pheochromocytoma, Paraganglioma, Pronchopulmonary, and other NETs	[^177^Lu]Lu-DOTATATE
Ryoo, H. G.	2023	Korea	6	7	Gastroenteropancreatic NETs	[^177^Lu]Lu-DOTATATE
Iqbal, S.	2024	United States	23	25	Gastroenteropancreatic and other NETs	[^177^Lu]Lu-DOTATATE
Shin, Y. ***	2024	Korea	43	64	Gastroenteropancreatic NETs	[^177^Lu]Lu-DOTATATE

* Patients with Minor Response (MR) were included in the evaluation. ** 24 patients could not be evaluated. *** 2 patients could not be evaluated. **** Complete Response (CR): Disappearance of all target lesions. Non-target lesions must also resolve or normalize. Partial Response (PR): At least a 30% decrease in the SLD of target lesions. Stable Disease (SD): No significant shrinkage (≥30%) or growth (≥20%) of the target lesions. Disease Control Rate (DCR).

**Table A4:** Disease control rates with radiolabeled somatostatin receptor Antagonists in patients with neuroendocrine tumors.

Study	Year	Country	Events (DCR = CR + PR + SD) *	Total	Neuroendocrine Tumor Types	Radiopharmaceuticals
Wild, D.	2014	Switzerland, Germany, United States	3	4	Bladder, Pulmonary, and Ileum NETs	[^177^Lu]Lu-DOTA-JR11
Reidy-Lagunes, D.	2019	United States, Germany	17	20	Gastroenteropancreatic, Pulmonary, and Kidney NETs	[^177^Lu]Lu-satoreotide tetraxetan
Baum, R. P.	2021	Germany, Singapore	40	47	Gastroenteropancreatic, Pulmonary, and other NETs	[^177^Lu]Lu-DOTA-LM3
Wild, D.	2023	Europe, Canada, Australia	36	38	Gastroenteropancreatic, Pulmonary, Paraganglioma, Pheochromocytoma and other NETs	[^177^Lu]Lu-satoreotide tetraxetan

* Complete Response (CR): Disappearance of all target lesions. Non-target lesions must also resolve or normalize. Partial Response (PR): At least a 30% decrease in the SLD of target lesions. Stable Disease (SD): No significant shrinkage (≥30%) or growth (≥20%) of the target lesions. Disease Control Rate (DCR).

## References

[1] Fortunati E., Bonazzi N., Zanoni L., Fanti S., Ambrosini V. (2023). Molecular imaging Theranostics of Neuroendocrine Tumors. Semin. Nucl. Med..

[2] Smith J., Barnett E., Rodger E.J., Chatterjee A., Subramaniam R.M. (2023). Neuroendocrine Neoplasms: Genetics and Epigenetics. PET Clin..

[3] Pavel M., Öberg K., Falconi M., Krenning E.P., Sundin A., Perren A., Berruti A. (2020). Gastroenteropancreatic neuroendocrine neoplasms: ESMO Clinical Practice Guidelines for diagnosis, treatment and follow-up. Ann. Oncol..

[4] Shah M.H., Goldner W.S., Benson A.B., Bergsland E., Blaszkowsky L.S., Brock P., Chan J., Das S., Dickson P.V., Fanta P. (2021). Neuroendocrine and Adrenal Tumors, Version 2.2021, NCCN Clinical Practice Guidelines in Oncology. J. Natl. Compr. Canc Netw..

[5] Nagtegaal I.D., Odze R.D., Klimstra D., Paradis V., Rugge M., Schirmacher P., Washington K.M., Carneiro F., Cree I.A. (2020). The 2019 WHO classification of tumours of the digestive system. Histopathology.

[6] Hope T.A., Pavel M., Bergsland E.K. (2022). Neuroendocrine Tumors and Peptide Receptor Radionuclide Therapy: When Is the Right Time?. J. Clin. Oncol..

[7] Ambrosini V., Kunikowska J., Baudin E., Bodei L., Bouvier C., Capdevila J., Cremonesi M., de Herder W.W., Dromain C., Falconi M. (2021). Consensus on molecular imaging and theranostics in neuroendocrine neoplasms. Eur. J. Cancer.

[8] Modlin I.M., Oberg K., Chung D.C., Jensen R.T., de Herder W.W., Thakker R.V., Caplin M., Delle Fave G., Kaltsas G.A., Krenning E.P. (2008). Gastroenteropancreatic neuroendocrine tumours. Lancet Oncol..

[9] Veenstra M.J., de Herder W.W., Feelders R.A., Hofland L.J. (2013). Targeting the somatostatin receptor in pituitary and neuroendocrine tumors. Expert. Opin. Ther. Targets.

[10] Rindi G., Mete O., Uccella S., Basturk O., La Rosa S., Brosens L.A.A., Ezzat S., de Herder W.W., Klimstra D.S., Papotti M. (2022). Overview of the 2022 WHO Classification of Neuroendocrine Neoplasms. Endocr. Pathol..

[11] Almeida C., Gervaso L., Frigè G., Spada F., Benini L., Cella C.A., Mazzarella L., Fazio N. (2024). The Role of Liquid Biopsy in Gastroenteropancreatic Neuroendocrine Neoplasms. Cancers.

[12] Alexander E.S., Ziv E. (2023). Neuroendocrine Tumors: Genomics and Molecular Biomarkers with a Focus on Metastatic Disease. Cancers.

[13] Fazio N., La Salvia A. (2023). Precision medicine in gastroenteropancreatic neuroendocrine neoplasms: Where are we in 2023?. Best. Pract. Res. Clin. Endocrinol. Metab..

[14] Harris P.E., Zhernosekov K. (2022). The evolution of PRRT for the treatment of neuroendocrine tumors; What comes next?. Front. Endocrinol..

[15] Grozinsky-Glasberg S., Grossman A.B., Korbonits M. (2008). The role of somatostatin analogues in the treatment of neuroendocrine tumours. Mol. Cell Endocrinol..

[16] Reubi J.C. (2003). Peptide receptors as molecular targets for cancer diagnosis and therapy. Endocr. Rev..

[17] Benali N., Ferjoux G., Puente E., Buscail L., Susini C. (2000). Somatostatin receptors. Digestion.

[18] Maecke H.R., Hofmann M., Haberkorn U. (2005). (68)Ga-labeled peptides in tumor imaging. J. Nucl. Med..

[19] Maccauro M., Follacchio G.A., Spreafico C., Coppa J., Seregni E. (2019). Safety and Efficacy of Combined Peptide Receptor Radionuclide Therapy and Liver Selective Internal Radiation Therapy in a Patient With Metastatic Neuroendocrine Tumor. Clin. Nucl. Med..

[20] Ambrosini V., Fani M., Fanti S., Forrer F., Maecke H.R. (2011). Radiopeptide imaging and therapy in Europe. J. Nucl. Med..

[21] Sakellis C., Jacene H.A. (2024). Neuroendocrine Tumors: Diagnostics. PET Clin..

[22] Santo G., Di Santo G., Virgolini I. (2024). Peptide Receptor Radionuclide Therapy of Neuroendocrine Tumors: Agonist, Antagonist and Alternatives. Semin. Nucl. Med..

[23] Marinova M., Mücke M., Fischer F., Essler M., Cuhls H., Radbruch L., Ghaei S., Conrad R., Ahmadzadehfar H. (2019). Quality of life in patients with midgut NET following peptide receptor radionuclide therapy. Eur. J. Nucl. Med. Mol. Imaging.

[24] Weckbecker G., Lewis I., Albert R., Schmid H.A., Hoyer D., Bruns C. (2003). Correction: Weckbecker et al. Opportunities in somatostatin research: Biological, chemical and therapeutic aspects. Nat. Rev. Drug Discov..

[25] Cescato R., Schulz S., Waser B., Eltschinger V., Rivier J.E., Wester H.J., Culler M., Ginj M., Liu Q., Schonbrunn A. (2006). Internalization of sst2, sst3, and sst5 receptors: Effects of somatostatin agonists and antagonists. J. Nucl. Med..

[26] Krebs S., O’Donoghue J.A., Biegel E., Beattie B.J., Reidy D., Lyashchenko S.K., Lewis J.S., Bodei L., Weber W.A., Pandit-Taskar N. (2020). Comparison of (68)Ga-DOTA-JR11 PET/CT with dosimetric (177)Lu-satoreotide tetraxetan ((177)Lu-DOTA-JR11) SPECT/CT in patients with metastatic neuroendocrine tumors undergoing peptide receptor radionuclide therapy. Eur. J. Nucl. Med. Mol. Imaging.

[27] Mallak N., Yilmaz B., Meyer C., Winters C., Mench A., Jha A.K., Prasad V., Mittra E. (2024). Theranostics in Neuroendocrine Tumors: Updates and Emerging Technologies. Curr. Probl. Cancer.

[28] Wild D., Fani M., Behe M., Brink I., Rivier J.E., Reubi J.C., Maecke H.R., Weber W.A. (2011). First clinical evidence that imaging with somatostatin receptor antagonists is feasible. J. Nucl. Med..

[29] Ginj M., Zhang H., Waser B., Cescato R., Wild D., Wang X., Erchegyi J., Rivier J., Mäcke H.R., Reubi J.C. (2006). Radiolabeled somatostatin receptor antagonists are preferable to agonists for in vivo peptide receptor targeting of tumors. Proc. Natl. Acad. Sci. USA.

[30] Wild D., Fani M., Fischer R., Del Pozzo L., Kaul F., Krebs S., Fischer R., Rivier J.E., Reubi J.C., Maecke H.R. (2014). Comparison of somatostatin receptor agonist and antagonist for peptide receptor radionuclide therapy: A pilot study. J. Nucl. Med..

[31] Zhu W., Cheng Y., Wang X., Yao S., Bai C., Zhao H., Jia R., Xu J., Huo L. (2020). Head-to-Head Comparison of (68)Ga-DOTA-JR11 and (68)Ga-DOTATATE PET/CT in Patients with Metastatic, Well-Differentiated Neuroendocrine Tumors: A Prospective Study. J. Nucl. Med..

[32] Zhu W., Jia R., Yang Q., Cheng Y., Zhao H., Bai C., Xu J., Yao S., Huo L. (2022). A prospective randomized, double-blind study to evaluate the diagnostic efficacy of (68)Ga-NODAGA-LM3 and (68)Ga-DOTA-LM3 in patients with well-differentiated neuroendocrine tumors: Compared with (68)Ga-DOTATATE. Eur. J. Nucl. Med. Mol. Imaging.

[33] Lin Z., Zhu W., Zhang J., Miao W., Yao S., Huo L. (2023). Head-to-Head Comparison of (68)Ga-NODAGA-JR11 and (68)Ga-DOTATATE PET/CT in Patients with Metastatic, Well-Differentiated Neuroendocrine Tumors: Interim Analysis of a Prospective Bicenter Study. J. Nucl. Med..

[34] Liu M., Ren C., Zhang H., Zhang Y., Huang Z., Jia R., Cheng Y., Bai C., Xu Q., Zhu W. (2024). Evaluation of the safety, biodistribution, dosimetry of [(18)F]AlF-NOTA-LM3 and head-to-head comparison with [(68)Ga]Ga-DOTATATE in patients with well-differentiated neuroendocrine tumors: An interim analysis of a prospective trial. Eur. J. Nucl. Med. Mol. Imaging.

[35] Grozinsky-Glasberg S., Barak D., Fraenkel M., Walter M.A., Müeller-Brand J., Eckstein J., Applebaum L., Shimon I., Gross D.J. (2011). Peptide receptor radioligand therapy is an effective treatment for the long-term stabilization of malignant gastrinomas. Cancer.

[36] Vaisman F., Rosado de Castro P.H., Lopes F.P., Kendler D.B., Pessoa C.H., Bulzico D.A., de Carvalho Leal D., Vilhena B., Vaisman M., Carneiro M. (2015). Is there a role for peptide receptor radionuclide therapy in medullary thyroid cancer?. Clin. Nucl. Med..

[37] Baum R.P., Kluge A.W., Kulkarni H., Schorr-Neufing U., Niepsch K., Bitterlich N., van Echteld C.J. (2016). [(177)Lu-DOTA](0)-D-Phe(1)-Tyr(3)-Octreotide ((177)Lu-DOTATOC) For Peptide Receptor Radiotherapy in Patients with Advanced Neuroendocrine Tumours: A Phase-II Study. Theranostics.

[38] Hörsch D., Ezziddin S., Haug A., Gratz K.F., Dunkelmann S., Miederer M., Schreckenberger M., Krause B.J., Bengel F.M., Bartenstein P. (2016). Effectiveness and side-effects of peptide receptor radionuclide therapy for neuroendocrine neoplasms in Germany: A multi-institutional registry study with prospective follow-up. Eur. J. Cancer.

[39] Nilica B., Waitz D., Stevanovic V., Uprimny C., Kendler D., Buxbaum S., Warwitz B., Gerardo L., Henninger B., Virgolini I. (2016). Direct comparison of (68)Ga-DOTA-TOC and (18)F-FDG PET/CT in the follow-up of patients with neuroendocrine tumour treated with the first full peptide receptor radionuclide therapy cycle. Eur. J. Nucl. Med. Mol. Imaging.

[40] Brabander T., van der Zwan W.A., Teunissen J.J.M., Kam B.L.R., Feelders R.A., de Herder W.W., van Eijck C.H.J., Franssen G.J.H., Krenning E.P., Kwekkeboom D.J. (2017). Long-Term Efficacy, Survival, and Safety of [(177)Lu-DOTA(0),Tyr(3)]octreotate in Patients with Gastroenteropancreatic and Bronchial Neuroendocrine Tumors. Clin. Cancer Res..

[41] Parghane R.V., Talole S., Prabhash K., Basu S. (2017). Clinical Response Profile of Metastatic/Advanced Pulmonary Neuroendocrine Tumors to Peptide Receptor Radionuclide Therapy with ^177^Lu-DOTATATE. Clin. Nucl. Med..

[42] Thapa P., Parghane R., Basu S. (2017). (177)Lu-DOTATATE Peptide Receptor Radionuclide Therapy in Metastatic or Advanced and Inoperable Primary Neuroendocrine Tumors of Rare Sites. World J. Nucl. Med..

[43] Demirci E., Kabasakal L., Toklu T., Ocak M., Şahin O.E., Alan-Selcuk N., Araman A. (2018). ^177^Lu-DOTATATE therapy in patients with neuroendocrine tumours including high-grade (WHO G3) neuroendocrine tumours: Response to treatment and long-term survival update. Nucl. Med. Commun..

[44] Carlsen E.A., Fazio N., Granberg D., Grozinsky-Glasberg S., Ahmadzadehfar H., Grana C.M., Zandee W.T., Cwikla J., Walter M.A., Oturai P.S. (2019). Peptide receptor radionuclide therapy in gastroenteropancreatic NEN G3: A multicenter cohort study. Endocr. Relat. Cancer.

[45] Sharma R., Wang W.M., Yusuf S., Evans J., Ramaswami R., Wernig F., Frilling A., Mauri F., Al-Nahhas A., Aboagye E.O. (2019). (68)Ga-DOTATATE PET/CT parameters predict response to peptide receptor radionuclide therapy in neuroendocrine tumours. Radiother. Oncol..

[46] Zandee W.T., Brabander T., Blažević A., Kam B.L.R., Teunissen J.J.M., Feelders R.A., Hofland J., de Herder W.W. (2019). Symptomatic and Radiological Response to ^177^Lu-DOTATATE for the Treatment of Functioning Pancreatic Neuroendocrine Tumors. J. Clin. Endocrinol. Metab..

[47] Adnan A., Sampathirao N., Basu S. (2019). Implications of fluorodeoxyglucose uptake in low-intermediate grade metastatic neuroendocrine tumors from peptide receptor radionuclide therapy outcome viewpoint: A semi-quantitative standardized uptake value-based analysis. World J. Nucl. Med..

[48] Beukhof C.M., Brabander T., van Nederveen F.H., van Velthuysen M.F., de Rijke Y.B., Hofland L.J., Franssen G.J.H., Fröberg L.A.C., Kam B.L.R., Visser W.E. (2019). Peptide receptor radionuclide therapy in patients with medullary thyroid carcinoma: Predictors and pitfalls. BMC Cancer.

[49] Prasad V., Srirajaskanthan R., Toumpanakis C., Grana C.M., Baldari S., Shah T., Lamarca A., Courbon F., Scheidhauer K., Baudin E. (2020). Lessons from a multicentre retrospective study of peptide receptor radionuclide therapy combined with lanreotide for neuroendocrine tumours: A need for standardised practice. Eur. J. Nucl. Med. Mol. Imaging.

[50] Satapathy S., Mittal B.R., Sood A., Sood A., Kapoor R., Gupta R. (2020). Peptide Receptor Radionuclide Therapy as First-Line Systemic Treatment in Advanced Inoperable/Metastatic Neuroendocrine Tumors. Clin. Nucl. Med..

[51] Graf J., Pape U.F., Jann H., Denecke T., Arsenic R., Brenner W., Pavel M., Prasad V. (2020). Prognostic Significance of Somatostatin Receptor Heterogeneity in Progressive Neuroendocrine Tumor Treated with Lu-177 DOTATOC or Lu-177 DOTATATE. Eur. J. Nucl. Med. Mol. Imaging.

[52] Hasan O.K., Ravi Kumar A.S., Kong G., Oleinikov K., Ben-Haim S., Grozinsky-Glasberg S., Hicks R.J. (2020). Efficacy of Peptide Receptor Radionuclide Therapy for Esthesioneuroblastoma. J. Nucl. Med..

[53] Huizing D.M.V., Aalbersberg E.A., Versleijen M.W.J., Tesselaar M.E.T., Walraven I., Lahaye M.J., de Wit-van der Veen B.J., Stokkel M.P.M. (2020). Early response assessment and prediction of overall survival after peptide receptor radionuclide therapy. Cancer Imaging.

[54] Kunikowska J., Zemczak A., Kołodziej M., Gut P., Łoń I., Pawlak D., Mikołajczak R., Kamiński G., Ruchała M., Kos-Kudła B. (2020). Tandem peptide receptor radionuclide therapy using (90)Y/(177)Lu-DOTATATE for neuroendocrine tumors efficacy and side-effects—polish multicenter experience. Eur. J. Nucl. Med. Mol. Imaging.

[55] Mirvis E., Toumpanakis C., Mandair D., Gnanasegaran G., Caplin M., Navalkissoor S. (2020). Efficacy and tolerability of peptide receptor radionuclide therapy (PRRT) in advanced metastatic bronchial neuroendocrine tumours (NETs). Lung Cancer.

[56] Parghane R.V., Naik C., Talole S., Desmukh A., Chaukar D., Banerjee S., Basu S. (2020). Clinical utility of (177) Lu-DOTATATE PRRT in somatostatin receptor-positive metastatic medullary carcinoma of thyroid patients with assessment of efficacy, survival analysis, prognostic variables, and toxicity. Head Neck.

[57] Pauwels E., Van Binnebeek S., Vandecaveye V., Baete K., Vanbilloen H., Koole M., Mottaghy F.M., Haustermans K., Clement P.M., Nackaerts K. (2020). Inflammation-Based Index and (68)Ga-DOTATOC PET-Derived Uptake and Volumetric Parameters Predict Outcome in Neuroendocrine Tumor Patients Treated with (90)Y-DOTATOC. J. Nucl. Med..

[58] Thiis-Evensen E., Poole A.C., Nguyen H.T., Sponheim J. (2020). Achieving objective response in treatment of non-resectable neuroendocrine tumors does not predict longer time to progression compared to achieving stable disease. BMC Cancer.

[59] Zemczak A., Kołodziej M., Gut P., Królicki L., Kos-Kudła B., Kamiński G., Ruchała M., Pawlak D., Kunikowska J. (2020). Effect of peptide receptor radionuclide therapy (PRRT) with tandem isotopes—[^90^Y]Y/[^177^Lu]Lu-DOTATATE in patients with disseminated neuroendocrine tumours depending on [^18^F]FDG PET/CT qualification in Polish multicentre experience—do we need [^18^F]FDG PET/CT for qualification to PRRT?. Endokrynol. Pol..

[60] Parghane R.V., Talole S., Basu S. (2021). (131)I-MIBG negative progressive symptomatic metastatic paraganglioma: Response and outcome with (177)Lu-DOTATATE peptide receptor radionuclide therapy. Ann. Nucl. Med..

[61] Severi S., Bongiovanni A., Ferrara M., Nicolini S., Di Mauro F., Sansovini M., Lolli I., Tardelli E., Cittanti C., Di Iorio V. (2021). Peptide receptor radionuclide therapy in patients with metastatic progressive pheochromocytoma and paraganglioma: Long-term toxicity, efficacy and prognostic biomarker data of phase II clinical trials. ESMO Open.

[62] Sistani G., Sutherland D.E.K., Mujoomdar A., Wiseman D.P., Khatami A., Tsvetkova E., Reid R.H., Laidley D.T. (2020). Efficacy of (177)Lu-Dotatate Induction and Maintenance Therapy of Various Types of Neuroendocrine Tumors: A Phase II Registry Study. Curr. Oncol..

[63] Teker F., Elboga U. (2021). Is SUVmax a useful marker for progression-free survival in patients with metastatic GEP-NET receiving (177)Lu-DOTATATE therapy?. Hell. J. Nucl. Med..

[64] Zandee W.T., Brabander T., Blažević A., Minczeles N.S., Feelders R.A., de Herder W.W., Hofland J. (2021). Peptide Receptor Radionuclide Therapy With ^177^Lu-DOTATATE for Symptomatic Control of Refractory Carcinoid Syndrome. J. Clin. Endocrinol. Metab..

[65] Almeamar H., Cullen L., Murphy D.J., Crowley R.K., Toumpanakis C., Welin S., O’Shea D., O’Toole D. (2022). Real-world efficacy of lutetium peptide receptor radionuclide therapy in patients with neuroendocrine tumours. J. Neuroendocrinol..

[66] Bongiovanni A., Nicolini S., Ibrahim T., Foca F., Sansovini M., Di Paolo A., Grassi I., Liverani C., Calabrese C., Ranallo N. (2022). (177)Lu-DOTATATE Efficacy and Safety in Functioning Neuroendocrine Tumors: A Joint Analysis of Phase II Prospective Clinical Trials. Cancers.

[67] Jiang Y., Liu Q., Wang G., Sui H., Wang R., Wang J., Zhang J., Zhu Z., Chen X. (2022). Safety and efficacy of peptide receptor radionuclide therapy with (177)Lu-DOTA-EB-TATE in patients with metastatic neuroendocrine tumors. Theranostics.

[68] Metser U., Eshet Y., Ortega C., Veit-Haibach P., Liu A., Rebecca K.S.W. (2022). The association between lesion tracer uptake on ^68^Ga-DOTATATE PET with morphological response to ^177^Lu-DOTATATE therapy in patients with progressive metastatic neuroendocrine tumors. Nucl. Med. Commun..

[69] Raj N., Coffman K., Le T., Do R.K.G., Rafailov J., Choi Y., Chou J.F., Capanu M., Dunphy M., Fox J.J. (2022). Treatment Response and Clinical Outcomes of Well-Differentiated High-Grade Neuroendocrine Tumors to Lutetium-177-DOTATATE. Neuroendocrinology.

[70] Zidan L., Iravani A., Oleinikov K., Ben-Haim S., Gross D.J., Meirovitz A., Maimon O., Akhurst T., Michael M., Hicks R.J. (2022). Efficacy and Safety of (177)Lu-DOTATATE in Lung Neuroendocrine Tumors: A Bicenter study. J. Nucl. Med..

[71] Sundlöv A., Gleisner K.S., Tennvall J., Ljungberg M., Warfvinge C.F., Holgersson K., Hallqvist A., Bernhardt P., Svensson J. (2022). Phase II trial demonstrates the efficacy and safety of individualized, dosimetry-based (177)Lu-DOTATATE treatment of NET patients. Eur. J. Nucl. Med. Mol. Imaging.

[72] Hentzen S., Mehta K., Al-Rajabi R.M.T., Saeed A., Baranda J.C., Williamson S.K., Sun W., Kasi A. (2023). Real world outcomes in patients with neuroendocrine tumor receiving peptide receptor radionucleotide therapy. Explor. Target. Antitumor Ther..

[73] Kepenek F., Kömek H., Can C., Kaplan İ., Altindağ S., Gündoğan C. (2023). The prognostic role of whole-body volumetric 68 GA-DOTATATE PET/computed tomography parameters in patients with gastroenteropancreatic neuroendocrine tumor treated with 177 LU-DOTATATE. Nucl. Med. Commun..

[74] Liu Q., Kulkarni H.R., Zhao T., Schuchardt C., Chen X., Zhu Z., Zhang J., Baum R.P. (2023). Peptide Receptor Radionuclide Therapy in Patients With Advanced Progressive Medullary Thyroid Cancer: Efficacy, Safety, and Survival Predictors. Clin. Nucl. Med..

[75] Mitjavila M., Jimenez-Fonseca P., Belló P., Pubul V., Percovich J.C., Garcia-Burillo A., Hernando J., Arbizu J., Rodeño E., Estorch M. (2023). Efficacy of [(177)Lu]Lu-DOTATATE in metastatic neuroendocrine neoplasms of different locations: Data from the SEPTRALU study. Eur. J. Nucl. Med. Mol. Imaging.

[76] Ryoo H.G., Suh M., Kang K.W., Lee D.W., Han S.W., Cheon G.J. (2023). Phase 1 Study of No-Carrier Added ^177^Lu-DOTATATE (SNU-KB-01) in Patients with Somatostatin Receptor-Positive Neuroendocrine Tumors: The First Clinical Trial of Peptide Receptor Radionuclide Therapy in Korea. Cancer Res. Treat..

[77] Iqbal S., Zhuang E., Raj M., Bahary N., Monga D.K. (2024). Long-term clinical outcomes of [(177)Lu]Lu-DOTATATE in patients with metastatic neuroendocrine tumors. Front. Oncol..

[78] Shin Y., Moon B.H., Ryoo B.Y., Chang H.M., Kim K.P., Hong Y.S., Kim T.W., Ryu J.S., Kim Y.I., Yoo C. (2024). Efficacy and Safety of Lu-177 DOTATATE Peptide Receptor Radionuclide Therapy in Patients with Unresectable or Metastatic Neuroendocrine Tumors in Korea. Target. Oncol..

[79] Reidy-Lagunes D., Pandit-Taskar N., O’Donoghue J.A., Krebs S., Staton K.D., Lyashchenko S.K., Lewis J.S., Raj N., Gönen M., Lohrmann C. (2019). Phase I Trial of Well-Differentiated Neuroendocrine Tumors (NETs) with Radiolabeled Somatostatin Antagonist (177)Lu-Satoreotide Tetraxetan. Clin. Cancer Res..

[80] Baum R.P., Zhang J., Schuchardt C., Müller D., Mäcke H. (2021). First-in-Humans Study of the SSTR Antagonist (177)Lu-DOTA-LM3 for Peptide Receptor Radionuclide Therapy in Patients with Metastatic Neuroendocrine Neoplasms: Dosimetry, Safety, and Efficacy. J. Nucl. Med..

[81] Wild D., Grønbæk H., Navalkissoor S., Haug A., Nicolas G.P., Pais B., Ansquer C., Beauregard J.M., McEwan A., Lassmann M. (2023). A phase I/II study of the safety and efficacy of [(177)Lu]Lu-satoreotide tetraxetan in advanced somatostatin receptor-positive neuroendocrine tumours. Eur. J. Nucl. Med. Mol. Imaging.

[82] Di Franco M., Zanoni L., Fortunati E., Fanti S., Ambrosini V. (2024). Radionuclide Theranostics in Neuroendocrine Neoplasms: An Update. Curr. Oncol. Rep..

[83] Ebner R., Sheikh G.T., Brendel M., Ricke J., Cyran C.C. (2025). ESR Essentials: Role of PET/CT in neuroendocrine tumors-practice recommendations by the European Society for Hybrid, Molecular and Translational Imaging. Eur. Radiol..

[84] Pomykala K.L., Hadaschik B.A., Sartor O., Gillessen S., Sweeney C.J., Maughan T., Hofman M.S., Herrmann K. (2023). Next generation radiotheranostics promoting precision medicine. Ann. Oncol..

[85] Gabriel M., Decristoforo C., Kendler D., Dobrozemsky G., Heute D., Uprimny C., Kovacs P., Von Guggenberg E., Bale R., Virgolini I.J. (2007). ^68^Ga-DOTA-Tyr3-octreotide PET in neuroendocrine tumors: Comparison with somatostatin receptor scintigraphy and CT. J. Nucl. Med..

[86] Theodoropoulou M., Stalla G.K. (2013). Somatostatin receptors: From signaling to clinical practice. Front. Neuroendocrinol..

[87] Park S., Parihar A.S., Bodei L., Hope T.A., Mallak N., Millo C., Prasad K., Wilson D., Zukotynski K., Mittra E. (2021). Somatostatin Receptor Imaging and Theranostics: Current Practice and Future Prospects. J. Nucl. Med..

[88] Binderup T., Knigge U., Loft A., Mortensen J., Pfeifer A., Federspiel B., Hansen C.P., Højgaard L., Kjaer A. (2010). Functional imaging of neuroendocrine tumors: A head-to-head comparison of somatostatin receptor scintigraphy, 123I-MIBG scintigraphy, and ^18^F-FDG PET. J. Nucl. Med..

[89] Strosberg J.R., Caplin M.E., Kunz P.L., Ruszniewski P.B., Bodei L., Hendifar A., Mittra E., Wolin E.M., Yao J.C., Pavel M.E. (2021). Correction: Strosberg at al. (177)Lu-Dotatate plus long-acting octreotide versus high-dose long-acting octreotide in patients with midgut neuroendocrine tumours (NETTER-1): Final overall survival and long-term safety results from an open-label, randomised, controlled, phase 3 trial. Lancet Oncol..

[90] Sheikhbahaei S., Sadaghiani M.S., Rowe S.P., Solnes L.B. (2021). Neuroendocrine Tumor Theranostics: An Update and Emerging Applications in Clinical Practice. AJR Am. J. Roentgenol..

[91] Salih S., Alkatheeri A., Alomaim W., Elliyanti A. (2022). Radiopharmaceutical Treatments for Cancer Therapy, Radionuclides Characteristics, Applications, and Challenges. Molecules.

[92] Fani M., Braun F., Waser B., Beetschen K., Cescato R., Erchegyi J., Rivier J.E., Weber W.A., Maecke H.R., Reubi J.C. (2012). Unexpected sensitivity of sst2 antagonists to N-terminal radiometal modifications. J. Nucl. Med..

[93] Bodei L., Weber W.A. (2018). Somatostatin Receptor Imaging of Neuroendocrine Tumors: From Agonists to Antagonists. J. Nucl. Med..

[94] Krebs S., Pandit-Taskar N., Reidy D., Beattie B.J., Lyashchenko S.K., Lewis J.S., Bodei L., Weber W.A., O’Donoghue J.A. (2019). Biodistribution and radiation dose estimates for (68)Ga-DOTA-JR11 in patients with metastatic neuroendocrine tumors. Eur. J. Nucl. Med. Mol. Imaging.

[95] Kunz P.L., Reidy-Lagunes D., Anthony L.B., Bertino E.M., Brendtro K., Chan J.A., Chen H., Jensen R.T., Kim M.K., Klimstra D.S. (2013). Consensus guidelines for the management and treatment of neuroendocrine tumors. Pancreas.

[96] Frilling A., Clift A.K. (2015). Therapeutic strategies for neuroendocrine liver metastases. Cancer.

